# The Altered Lipid Composition and Key Lipid Metabolic Enzymes in Thiacloprid-Resistant *Myzus persicae*, with Special Attention Paid to the Function of *MpTHEM6a*

**DOI:** 10.3390/ijms252212112

**Published:** 2024-11-11

**Authors:** Jinfeng Hu, Wenhua Rao, Feng Chen, Xianzhi Zhou, Jun Wang, Lei Lin, Guocheng Fan

**Affiliations:** Fujian Engineering Research Center for Green Pest Management, Key Laboratory for Monitoring and Integrated Management of Crop Pests, Institute of Plant Protection, Fujian Academy of Agricultural Sciences, Fuzhou 350003, China

**Keywords:** *Myzus persicae*, thiacloprid resistance, lipid composition, lipid metabolic enzymes, MpTHEM6a

## Abstract

Neonicotinoid resistance is increasingly prevalent in the agricultural pest *Myzus persicae*. Lipids play a critical role in insect defense systems, but their contribution to insect neonicotinoid resistance is disregarded. We conducted metabolomics and transcriptomics studies on *M. persicae* thiacloprid-resistant (THG-R) and -susceptible (FFJ-S) populations. A total of 149 lipid metabolites were identified, with 90 upregulated and 59 downregulated in THG-R compared to in FFJ-S. Metabolites in the arachidonic acid (AA) pathway substantially varied between THG-R and FFJ-S. For example, arachidonic acid, (±)11-HETE, and prostaglandin B1 were significantly upregulated, while prostaglandin A1, tetranor-PGDM, 8,15-diHETE, and (±)11(12)-EET were significantly decreased in THG-R. Transcriptomics profiles and qPCR indicated that lipid metabolic enzymes, including fatty acid synthase (FAS), the elongase of very-long-chain fatty acids (ELO), fatty acid desaturase (FAD), and phospholipase (PL) genes, were not overexpressed in THG-R. Among the twelve thioesterase genes, only *MpTHEM6a* was significantly upregulated in THG-R. Knocking down the expression of *MpTHEM6a* in THG-R significantly increased the toxicity of the three neonicotinoids, reduced the lifespan of adults, and decreased the number of nonviable nymphs produced by female adults. The metabolites AA, (±)11-HETE, and prostaglandin B1 are potential biomarkers in neonicotinoid-resistant *M. persicae*. *MpTHEM6a* may become a potential target for combating neonicotinoid-resistant *M. persicae*.

## 1. Introduction

The green peach aphid *Myzus persicae* (Sulzer) is one of the most economically important agricultural pests. It can feed on more than 400 plant species belonging to 50 families, including potato, tobacco, and eggplant. The GPA is notorious because it can act as a vector and transmit 115 different plant viruses, accounting for 67.7% of aphid vector viruses [1]. Insecticides are the foundation of the management of the GPA in China and other countries. Neonicotinoids, which target the nicotinic acetylcholine receptors (nAChRs) of insects, mainly include acetamiprid, clothianidin, dinotefuran, imidacloprid, thiacloprid, and thiamethoxam, and they have become the most widely used insecticides for the control of *M. persicae* [2]. However, resistance to neonicotinoids in *M. Persicae* has been reported worldwide and is a growing concern [3,4,5]. Knowledge on the mechanisms of resistance of *M. Persicae* to neonicotinoids can help in establishing rational control strategies to delay the development of resistance and extend the service life of neonicotinoids.

For several decades, research on neonicotinoid resistance in pests has mainly focused on target-site and metabolic resistance [6,7]. The overexpression of metabolic enzymes, such as P450 genes in resistant pests, plays a crucial role in the enhanced detoxification of neonicotinoids. However, the evolution of insecticide resistance in pests is a complex genetic phenomenon, and a large number of enzymes are involved. Enzymes related to lipid metabolism have been proven to be associated with insecticide resistance in pests. For instance, *Anophleles arabiensis* and *Anopheles gambiae* populations that overexpress CYP4G16 and CYP4G17 show a higher deposition of cuticular hydrocarbons, which are linked to both resistance to insecticides and improved mating success [8,9,10]. Carboxylesterases derived from the αEsterase gene cluster, including αE7 from *Lucilia cuprina* and *Drosophila melanogaster* [11], have a crucial physiological function in lipid metabolism and are involved in the biodegradation of organophosphate (OP) insecticides. However, the function of some insect lipid metabolic enzymes in insecticide resistance is overlooked.

In the healthy nervous system, insect lipids, similar to vertebrate lipids, play a role in hormone synthesis and coordinate their metabolism with detoxification enzymes and antimicrobial peptides. The dysfunction of lipid metabolism enzymes disrupts normal lipid metabolism, thus causing functional disorders in insect bodies. Many studies have examined the effects of pesticides on lipids and their metabolism in non-target insects, such as *Aphis mellifera* and *D. melanogaster*, and it has been found that they mainly alter the lipid constituents of cells, leading to tissue and organ function disorders. A previous study found that low doses of spinosad, a microbial insecticide targeting the α6 subunit of nAChRs, triggered lipid dysregulation in *D. melanogaster*, as well as increasing lipid stores in the fat body and reducing lipid droplet numbers in the Malpighian tubules. This also indicates that the knockout of α6 from the membrane precipitated by spinosad exposure in wild-type flies leads to their death [12]. Another study found that exposure to neonicotinoid pesticides alters the lipid composition in insects. Lipid metabolite ratios significantly differed between control and imidacloprid-exposed *A. mellifera* [13]. Additionally, Cook reported that high-dose clothianidin, a neonicotinoid, reduced the lipid content in bees [14]. Furthermore, after exposure to neonicotinoids, a reduction in lipid peroxidation (LPO) was observed in many non-target insects, such as *Chironomus riparius* and *A. mellifera* [15,16].

**Figure 1:**
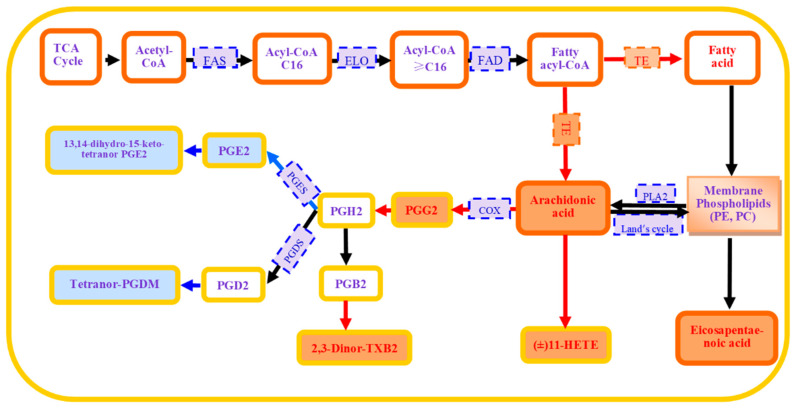
Fatty acid metabolism in thiacloprid-resistant *M. persicae* (THG-R) with the major metabolites and key enzymes. The individual metabolites are described in detail in the text. A jacinth background indicates upregulated metabolites and a sky-blue background indicates downregulated metabolites in the THG-R population compared with the susceptible population (FFJ-S). The red arrows indicate an increase, and the green arrows indicate a reduction. A white background indicates that the specific metabolite compound is unclear or has not been identified. *FAS*: fatty acid synthase; ELO: the elongase of very-long-chain fatty acids; FAD: fatty acid desaturase; TE: thioesterase; PLA2: phospholipase A2; COX: cyclooxygenase; PGDS: prostaglandin D synthase; PGES: prostaglandin E synthase.

As the second largest component in insects, lipids comprise a chemically diverse group of fatty acids, glycolipids, glycerophospholipids, sphingolipids, sterols, and phenols [17]. These lipids are indispensable for maintaining the normal physiological functions of insects. Lipid metabolism involves many enzymes which play a crucial role in maintaining the balance of lipids in insects. For example, fatty acids (FAs) are a type of aliphatic hydrocarbon chain with a carboxyl group at one end. Their biosynthesis is a complex multi-step reaction process starting from acetyl-CoA (Figure 1), mainly carried out by enzymes such as fatty acid synthase (FAS), the elongase of very-long-chain fatty acids (ELO), fatty acid desaturase (FAD), and thioesterase [18]. Additionally, other enzymes are involved in fatty acid hydrolysis, such as fatty acyl-CoA reductase (FAR), which converts fatty acid to fatty aldehyde. Furthermore, phospholipases, such as phospholipase A2 (PLA2), can catalyze phospholipids (PLs) to release arachidonic acid (AA), a fatty acid which is usually used for the biosynthesis of eicosanoids in insects.

Previous studies have mostly focused on the short-term effects of insecticide exposure on insect lipid composition and metabolism. However, insecticide resistance in insects evolves under the selective pressure of insecticides. During this process, insects adapt to the constantly changing environment, leading to the emergence of new traits. Apart from studies on specific lipid composition and metabolism, other aspects of lipid composition and metabolism related to insecticide resistance have been rarely investigated. The emergence of new research methods, such as metabolomics and transcriptomics, has helped in clarifying lipid metabolism associated with insect resistance to insecticides. In this study, we obtained *M. persicae* with high thiacloprid resistance through continuous screening with thiacloprid in the laboratory. Metabolomics and transcriptomics were applied to determine the differences in lipid composition and related metabolic enzymes between thiacloprid-resistant and -susceptible *M. persicae*. We also conducted functional validation to provide a reference for understanding the role of lipid metabolism in *M. persicae* resistance to neonicotinoid insecticides and for identifying new targets for insecticides.

## 2. Results

### 2.1. Comparison of Lipid Metabolites Between THG-R and FFJ-S Populations

**Figure 2:**
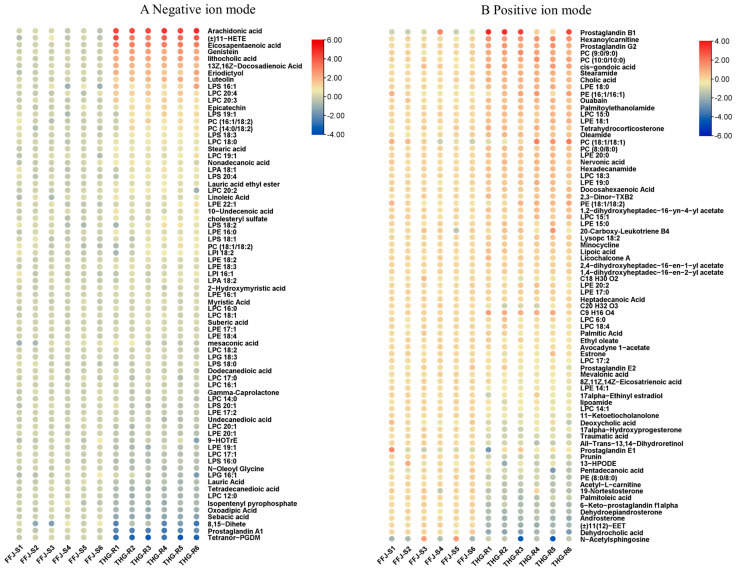
A heatmap of lipid metabolite profiles, comparing the THG-R and FFJ-S populations. PC: phosphatidylcholine; LPC: lysophosphatidylcholine; PE: phosphatidylethanolamine; LPE: lyso-phosphatidylethanolamine; LPI: lyso-phosphatidylglycerol; LPS: lysophosphatidylserine; EET: epoxyeicosatrienoic acid; AA: arachidonic acid; HETE: hydroxyeicosatetraene; HPODE: hydroperoxy-9*Z*,11*E*octadecadienoic acid.

In both negative and positive ion modes, metabolomics revealed a total of 148 lipid metabolites in the aphids (Figure 2). The lipids identified in the negative ion mode were classified into five categories: fatty acids, glycerophospholipids, polyketides, prenol lipids, and sterols. Glycerophosphocholines were the predominant compounds, with 17 distinct species, followed by fatty acids and conjugates, with 16 metabolites (Supplement Figure S1). The lipid metabolites identified in the positive ion mode were further classified into six categories: fatty acids, glycerophospholipids, polyketides, prenol lipids, sphingolipids, and steroids. Within these subclasses, “fatty acids and conjugates” represented the largest group, with 13 metabolites, closely followed by glycerophosphocholines with 12 metabolites.

**Figure 3:**
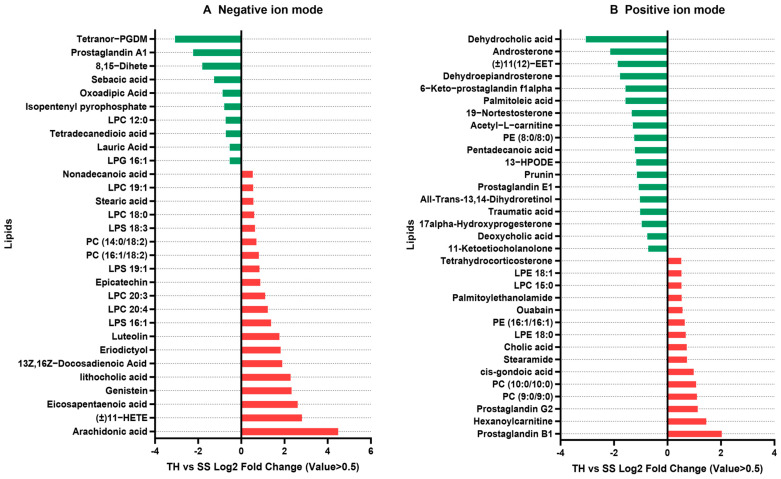
The top different lipids (|Log2Fold Change| > 0.5) between the THG-R and FFJ-S populations of *M. persicae*.

A quantitative analysis revealed differences in the lipid metabolites between the THG-R and FFJ-S populations (Figure 2). Of all the lipid metabolites, 90 were upregulated and 58 were downregulated in the THG-R population (Figure 2). The metabolites with the most significant differences (Log2Fold ≥ 0.5) are shown in Figure 3. The results indicate that compared with the lipid metabolites in FFJ-S, in the negative ion mode, the resistant population THG-R had significantly increased levels of five lipids (Log2Fold > 2), namely, arachidonic acid, (±)11-HETE (Log2Fold = 3.6, *p* < 0.0001), eicosapentaenoic acid (Log2Fold = 3.2, *p* < 0.0001), genistein, and lithocholic acid. In the positive ion mode, the resistant population THG-R had significantly increased levels of five lipids (Log2Fold > 1), namely, prostaglandin B1, hexanoylcarnitine, prostaglandin G2, PC (9:0/9:0), and PC (10:0/10:0). In the negative ion mode, the resistant population THG-R had significantly decreased levels of five lipids (Log2Fold < −0.9), namely, oxoadipic acid, sebacic acid, 8,15-dihete, prostaglandin A1, and tetrahydro-PGDM. In the positive ion mode, the resistant population THG-R had significantly decreased levels of five lipids (Log2Fold < −1.5), namely, 6-keto-prostaglandin f1 alpha, dehydroepiandrosterone, androsterone, dehydrocholic acid, and N-acetylsphingosine. Among the top 20 lipid metabolites with the greatest differences, 6 were related to prostaglandin metabolism. Those significantly upregulated in THG-R were arachidonic acid (Log2Fold = 4.49, *p* < 0.0001), prostaglandin B1 (Log2Fold = 2.03, *p* < 0.001), and prostaglandin G2 (Log2Fold = 1.45, *p* < 0.0001), while those significantly downregulated were prostaglandin A1 (Log2Fold = −2.03, *p* < 0.0001), 8,5-diHETE (Log2Fold = −3.07, *p* < 0.01), tetranor-PGDM (Log2Fold = −3.07, *p* < 0.0001), and 6-keto-prostaglandin f1 alpha (Log2Fold = −1.57, *p* < 0.0001). This finding indicates a significant difference between the two groups in terms of the AA metabolic pathways associated with prostaglandins (Figure 1).

Regarding glycerophospholipids, a total of 29 compounds belonged to phosphatidylcholine (PC) and lysophosphatidylcholine (LPC), with 19 upregulated and 10 downregulated metabolites. Regarding phosphatidylethanolamine (PE) and lysophosphatidylethanolamine (LPE), 21 metabolites (i.e., 16 up- and 5 downregulated metabolites) were included (Figure 2). The significantly upregulated PEs and LPEs in THG-R included LPE 18:0 (Log2Fold = 0.68, *p* < 0.0001), PE (16:1/16:1) (Log2Fold = 0.65, *p* = 0.031), LPE 18:1 (Log2Fold = 0.52, *p* < 0.01), LPE 20:0 (Log2Fold = 0.47, *p* < 0.0001), LPE 19:0 (Log2Fold = 0.43, *p* < 0.001), LPE 22:1 (Log2Fold = 0.38, *p* = 0.031), PE (18:1/18:2) (Log2Fold = 0.37, *p* = 0.074), LPE 16:0 (Log2Fold = 0.36, *p* < 0.01), LPE 15:0 (Log2Fold = 0.29, *p* = 0.028), LPE 18:2 (Log2Fold = 0.25, *p* < 0.0001), LPE 18:3 (Log2Fold = 0.23, *p* < 0.001), and LPE 16:1 (Log2Fold = 0.13, *p* < 0.001). In addition, a few PC and LPC metabolites were also significantly increased in THG-R, including LPC 20:4 (Log2Fold = 1.23, *p* < 0.0001), LPC 20:3 (Log2Fold = 1.11, *p* < 0.0001), PC (9:0/9:0) (Log2Fold = 1.1, *p* < 0.0001), PC (10:0/10:0) (Log2Fold = 1.07, *p* < 0.0001), PC (16:1/18:2) (Log2Fold = 0.81, *p* < 0.01), PC (14:0/18:2) (Log2Fold = 0.7, *p* < 0.001), LPC 18:0 (Log2Fold = 0.59, *p* < 0.0001), LPC 19:1 (Log2Fold = 0.55, *p* < 0.001), LPC 15:0 (Log2Fold = 0.53, *p* < 0.0001), and PC (8:0/8:0) (Log2Fold = 0.49, *p* < 0.0001). Five LPE metabolites (LPE 17:2 (Log2Fold = −0.21, *p* < 0.01), LPE 20:1 (Log2Fold = −0.34, *p* < 0.0001), LPE 14:1 (Log2Fold = −0.42, *p* < 0.001), LPE 19:1 (Log2Fold = −0.43, *p* = 0.011), PE (8:0/8:0) (Log2Fold = −1.22, *p* < 0.0001)), and five LPC metabolites (LPC 14:0 (Log2Fold = −0.17, *p* = 0.012), LPC 20:1 (Log2Fold = −0.3, *p* < 0.01), LPC 17:2 (Log2Fold = −0.26, *p* < 0.001), LPC 17:1 (Log2Fold = −0.43, *p* < 0.0001), and LPC 14:1 (Log2Fold = −0.45, *p* < 0.0001)) exhibited a significant decrease in THG-R.

### 2.2. Characteristics and Expression Patterns of MpFASs in THG-R and FFJ-S Populations

**Figure 4:**
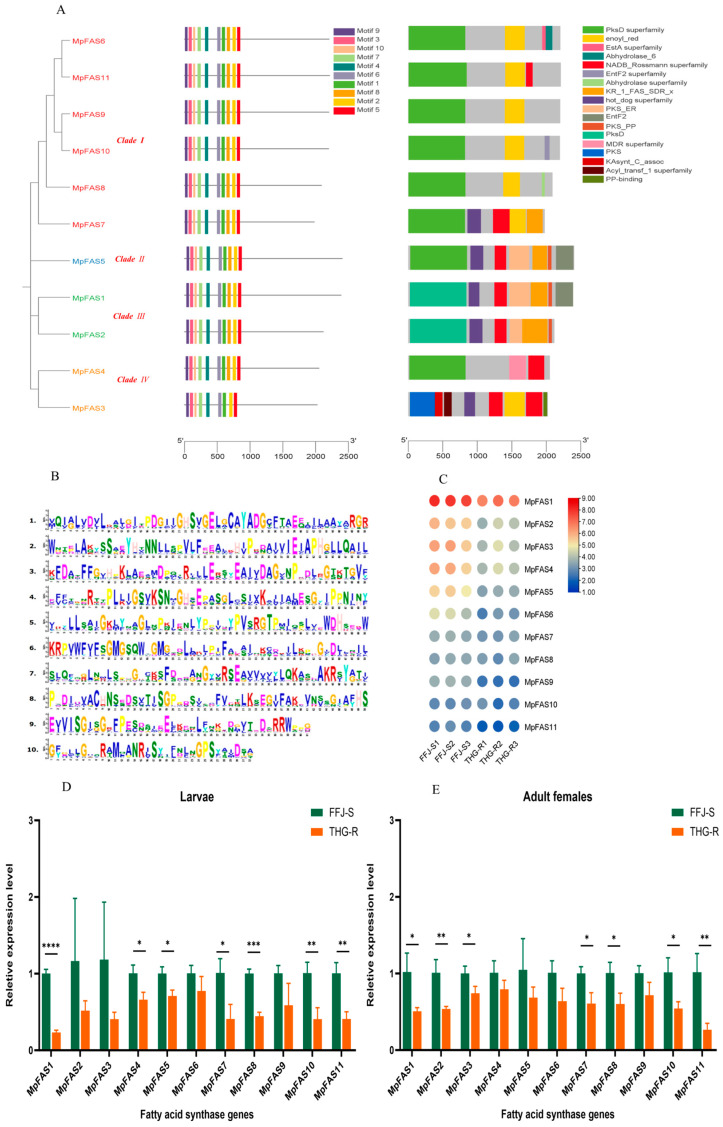
Phylogenetic trees and the expression profiles of *MpFAS*s in the THG-R and FFJ-S populations. (**A**) The constructed phylogenetic tree, gene structure, and conserved protein structure of *MpFAS*s. (**B**) An image of the motif sequences in *MpFAS*s. (**C**) A heatmap analysis of *MpFAS* transcripts in the THG-R and FFJ-S populations. The mRNA levels, represented by normalized Log2 (FPKM) values, are shown in the gradient heatmap, with colors ranging from blue (low expression) to red (high expression). (**D**) Quantitative expression of MpFASs determined via qRT-PCR in the second-instar larvae of the THG-R and FFJ-S populations. (**E**) Quantitative expression of *MpFAS*s determined via qRT-PCR in the adult females of the THG-R and FFJ-S populations. The expression levels were normalized to β-actin genes. The significant differences are marked by asterisks: * at the 0.05 level; ** at the 0.01 level; *** at the 0.001 level; **** at the 0.0001 level.

A total of 11 candidate FAS genes were identified in the aphid genome. A phylogenetic tree analysis indicated that these 11 *MpFAS* genes belonged to four categories, with most being in Clade Ⅰ, totaling 6, followed by Clade Ⅳ and Ⅲ with 2 each and Clades Ⅱ with only 1 (Figure 4A). Except for the gene MpFAS3, which contained 9 motifs, the other *MpFASs* contained 10 motifs, including the functional catalytic motif “GSVKS” (motif 4) (Figure 4A,B). An analysis of the gene domains revealed that there were 18 domains among the 11 *MpFAS* genes. Additionally, eight MpFAS genes contained the “PksD superfamily” and “NADB_Rossmann superfamily” domains, and seven genes contained the “PksD” domain (Figure 4A).

Based on the transcriptomic data, a transcriptional expression analysis of the 11 *MpFAS* genes in the peach aphid was conducted, and it revealed that the expression level of *MpFAS1* was the highest, while that of MpFAS11 was the lowest (Figure 4C). The transcriptomic data also indicated that the expression levels of the *MpFAS* genes in the THG-R population were lower than those in the FFJ-S population. The fluorescence quantitative PCR results showed that among the second-instar nymphs, the expression levels of seven genes, namely, *MpFAS1*, *MpFAS4*, *MpFAS5*, *MpFAS7*, *MpFAS8*, *MpFAS10*, and *MpFAS11*, were significantly lower in the THG-R population than in the FFJ-S population (Figure 4D). Among the adult females, the expression levels of seven genes, namely, *MpFAS1*, *MpFAS2*, *MpFAS3*, *MpFAS7*, *MpFAS8*, *MpFAS10*, and *MpFAS11*, were significantly lower in the THG-R population than in the FFJ-S population (Figure 4E).

### 2.3. Characteristics and Expression Patterns of MpELOs in THG-R and FFJ-S Populations

**Figure 5:**
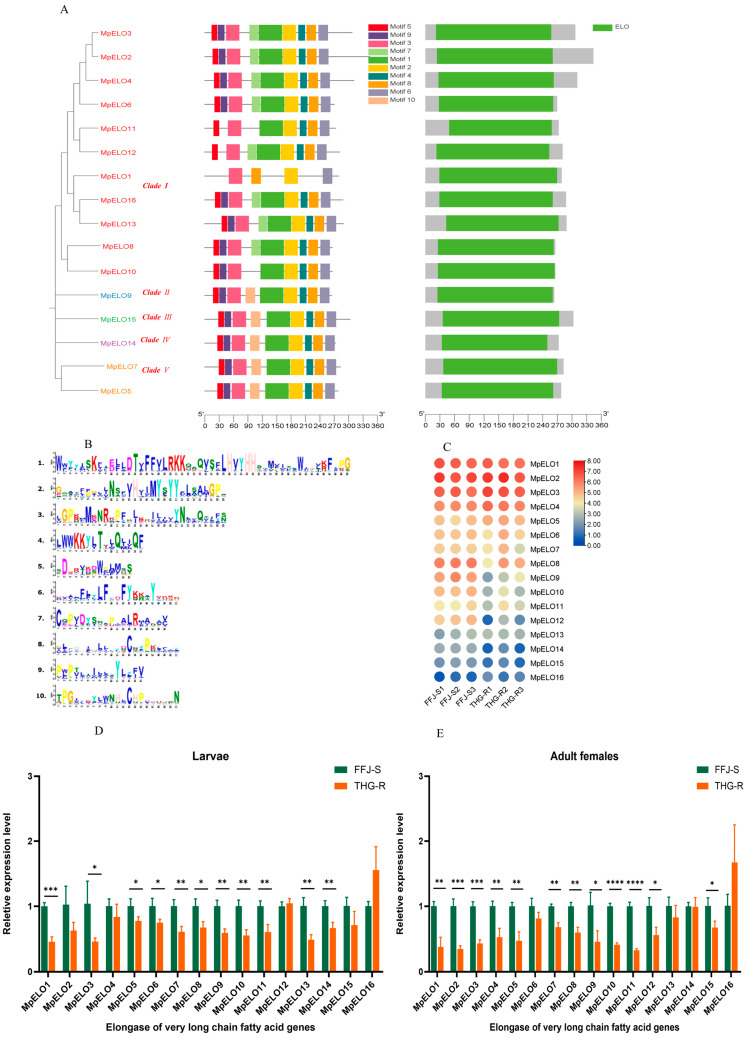
Phylogenetic trees and their expression profiles of *MpELO*s in the THG-R and FFJ-S populations. (**A**) The constructed phylogenetic tree, gene structure, and conserved protein structure of *MpELO*s. (**B**) An image of the motif sequences in *MpELO*s. (**C**) A heatmap analysis of *MpELO*s transcripts in the THG-R and FFJ-S populations. The mRNA levels, represented by normalized Log2 (FPKM) values, are shown in the gradient heatmap, with colors ranging from blue (low expression) to red (high expression). (**D**) Quantitative expression of *MpELO*s by qRT-PCR in the 2nd-instar larvae of the THG-R and FFJ-S populations. (**E**) Quantitative expression of *MpELO*s by qRT-PCR in the adult females of the THG-R and FFJ-S populations. The expression levels were normalized to β-actin genes. The significant differences are marked by asterisks: * at the 0.05 level; ** at the 0.01 level; *** at the 0.001 level; **** at the 0.0001 level.

We detected 16 *MpELO* genes in the aphid genome. A phylogenetic tree analysis indicated that these 16 *MpELO* genes belonged to five categories, with most being in Clade I, totaling 11, followed by Clade V with 2 and Clades II, III, and IV with only 1 each (Figure 5A). Among the 16 MpELO genes, the minimum number of motifs contained was 10, such as KXXEXXDT, HXXMYXYY, TXXQXXQ, and HXXHH (motif 1), a histidine-box motif that is conserved in all elongases (Figure 5A,B). An analysis of the gene domains revealed that all 16 *MpELO* genes contained only the “ELO” domain (Figure 5A).

Using FPKM as the standard, the transcriptomic data indicated that among the 16 *MpELO* genes in the peach aphid, the expression levels of *MpELO1*, *MpELO2*, *MpELO3*, and *MpELO4* were all relatively high, while the expression level of *MpELO11* was the lowest (Figure 5C). The transcriptomic data also showed that the expression levels of the *MpELO* genes in the THG-R population were lower than those in the FFJ-S population. The fluorescence quantitative PCR results revealed that among the second-instar nymphs, the expression levels of 11 genes, namely, *MpELO1*, *MpELO3*, *MpELO5*, *MpELO6*, *MpELO7*, *MpELO8*, *MpELO9*, *MpELO10*, *MpELO11*, *MpELO13*, and *MpELO14*, were significantly lower in the THG-R population than in the FFJ-S population (Figure 5D). Among the adult females, the expression levels of 12 genes, namely, *MpELO1*, *MpELO2*, *MpELO3*, *MpELO4*, *MpELO5*, *MpELO7*, *MpELO8*, *MpELO9*, *MpELO10*, *MpELO11*, *MpELO12*, and *MpELO14*, were significantly lower in the THG-R population than in the FFJ-S population (Figure 5E). The differences in the expressions of the other *MpELO* genes were not significant.

### 2.4. Characteristics and Expression Patterns of MpFADs in THG-R and FFJ-S Populations

**Figure 6:**
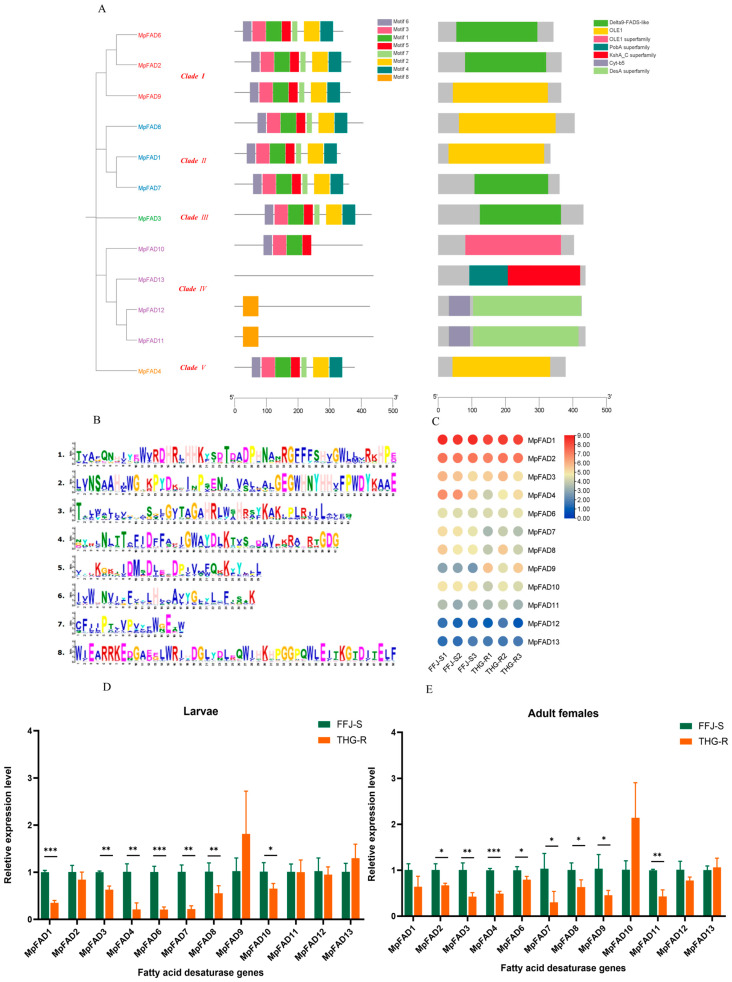
Phylogenetic trees and their expression profiles of *MpFAD*s in the THG-R and FFJ-S populations. (**A**) The constructed phylogenetic tree, gene structure, and conserved protein structure of MFADs. (**B**) An image of the motif sequences in *MpFAD*s. (**C**) A heatmap analysis of *MpFAD* transcripts in the THG-R and FFJ-S populations; the mRNA levels, represented by normalized Log2 (FPKM) values, are shown in the gradient heatmap, with colors ranging from blue (low expression) to red (high expression). (**D**) Quantitative expression of *MpFAD*s by qRT-PCR in the 2nd-instar larvae of the THG-R and FFJ-S populations. (**E**) Quantitative expression of *MpFAD*s by qRT-PCR in the adult females of the THG-R and FFJ-S populations. The expression levels were normalized to β-actin genes. The significant differences are marked by asterisks: * at the 0.05 level; ** at the 0.01 level; *** at the 0.001 level.

A total of 12 *MpFAD* genes were detected in the aphid genome and classified into five clades, with most being in Clade IV, totaling 4, followed by Clades I and II with 2 each and by Clades III and V with only 1 each (Figure 6A). Among the 12 *MpFAD* genes, the minimum number of motifs contained was eight, with *MpFAD11* and *MpFAD12* containing only motif 8 (Figure 6A,B). An analysis of the gene domains revealed that among the 11 *MpFAD* genes, 9 contained only one domain, and 4 contained the “Delta9-FADS-like” and “OLE1” domains. Unlike the other genes, *MpFAD11* and *MpFAD12* shared two domains: “Cyt-b5” and “DesA superfamily” (Figure 6A).

Based on the transcriptomic data, a transcriptional expression analysis of the 12 MpFAD genes in the peach aphid showed that the expression levels of *MpFAD1* and *MpFAD2* were the highest, while the expression level of *MpFAD11* was the lowest (Figure 6C). The transcriptomic data also indicated that the expression levels of the *MpFAD* genes in the THG-R population were lower than those in the FFJ-S population. The fluorescence quantitative PCR results revealed that among the second-instar nymphs, the expression levels of seven genes, namely, *MpFAD1*, *MpFAD3*, *MpFAD4*, *MpFAD6*, *MpFAD7*, *MpFAD8*, and *MpFAD10*, were significantly lower in the THG-R population than in the FFJ-S population (Figure 6D). Among the adult females, the expression levels of eight genes, namely, *MpFAD2*, *MpFAD3*, *MpFAD4*, *MpFAD6*, *MpFAD7*, *MpFAD8*, *MpFAD9*, and *MpFAD11*, were significantly lower in the THG-R population than in the FFJ-S population (Figure 6E). The differences in the expressions of the other *MpFAD* genes were not significant.

### 2.5. Characteristics and Expression Patterns of MpTEs in THG-R and FFJ-S Populations

**Figure 7:**
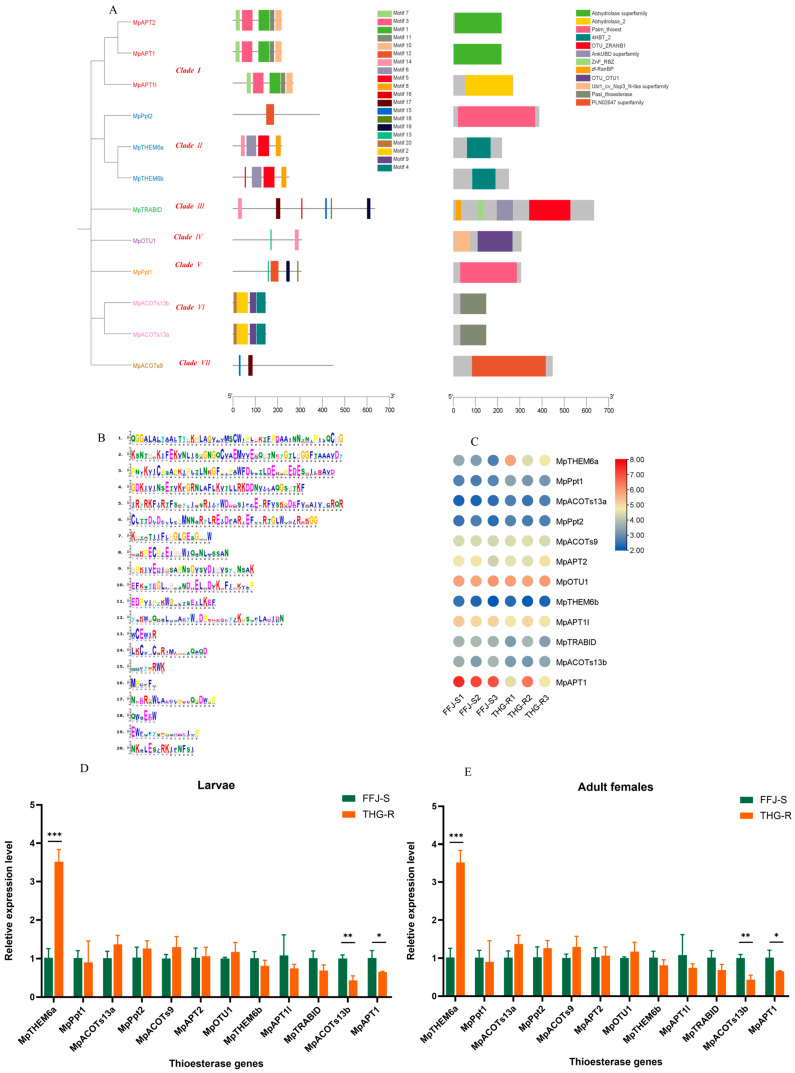
Phylogenetic trees and their expression profiles of *MpTE*s in the THG-R and FFJ-S populations. (**A**) The constructed phylogenetic tree, gene structure, and conserved protein structure of *MpFAD*s. (**B**) An image of the motif sequences in thioesterases of *M. persicae*. (**C**) A heatmap analysis of MpTE transcripts in the THG-R and FFJ-S populations. The mRNA levels, represented by normalized Log2 (FPKM) values, are shown in the gradient heatmap, with colors ranging from blue (low expression) to red (high expression). (**D**) Quantitative expression of *MpTE*s by qRT-PCR in the 2nd-instar larvae of the THG-R and FFJ-S populations. (**E**) Quantitative expression of *MpTE*s by qRT-PCR in the adult females of the THG-R and FFJ-S populations. The expression levels were normalized to β-actin genes. The significant differences are marked by asterisks: * at the 0.05 level; ** at the 0.01 level; *** at the 0.001 level.

We identified 12 thioesterase genes (Figure 7). A phylogenetic analysis revealed that these genes belonged to seven distinct clades encompassing prominent thioesterase families, such as acyl-protein thioesterases (three members), *THEM6* genes (two members), ubiquitin thioesterases (two members), and acyl-CoA thioesterases (two members) (Figure 7A). The smallest number of motifs in these thioesterase genes was found to be 20 (Figure 7B). A domain architecture analysis further revealed that ten *MpTEs* only possessed a single domain, which belonged to the “Abhydrolase superfamily”, “Abhydrolase_2”, “Palm thioesterase”, “4HBT_2”, “Paal_thioesterase”, and “PLN02647 superfamily” categories (Figure 7A).

The transcriptomic data of *M. persicae* revealed the transcriptional expression profiles of the 12 thioesterase genes, indicating that *MpAPT1* and *MpOPT1* exhibited higher expression levels, whereas *MpACOTs13a*, *MpPpt2*, and *MpTHEM6b* showed lower expression levels (Figure 7C). The transcriptomic data also demonstrated that the expression levels of *MpTHEM6a* and *MpPpt1* were higher in the THG-R population than in the FFJ-S population, while the expression levels of *MpAPT1l*, *MpTRABID*, *MpACOTs13b*, and *MpAPT1* were lower in the THG-R population than in the FFJ-S population. The quantitative fluorescence PCR results further confirmed that among the second-instar nymphs and female adults, the expression level of *MpTHEM6a* was significantly higher in the THG-R population than in the FFJ-S population, whereas the expression levels of *MpACOTs13b* and *MpAPT1* were significantly lower in the THG-R population than in the FFJ-S population (Figure 7D,E).

### 2.6. Characteristics and Expression Patterns of MpPLs in THG-R and FFJ-S Populations

**Figure 8:**
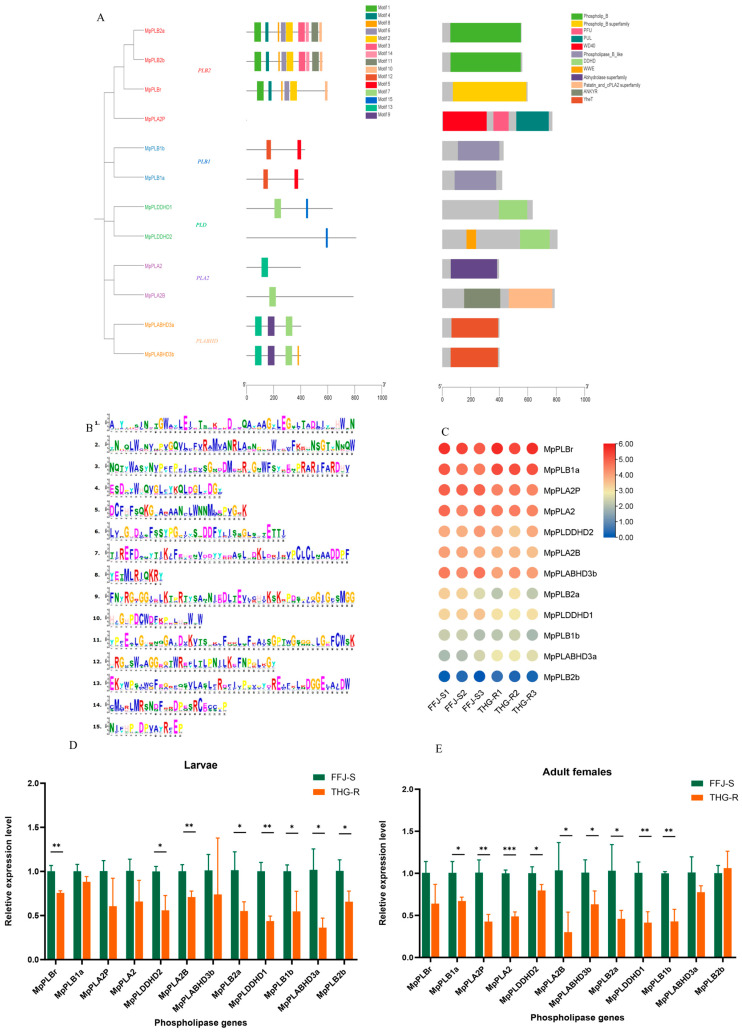
Phylogenetic trees and their expression profiles of *MpPL*s in the THG-R and FFJ-S populations. (**A**) The constructed phylogenetic tree, gene structure, and conserved protein structure of *MpPLs*. (**B**) An image of the motif sequences in *MpPLs*. (**C**) A heatmap analysis of *MpPLs* transcripts in the THG-R and FFJ-S populations. The mRNA levels, represented by normalized Log2 (FPKM) values, are shown in the gradient heatmap, with colors ranging from blue (low expression) to red (high expression). (**D**) Quantitative expression of MpPLs by qRT-PCR in the 2nd-instar larvae of the THG-R and FFJ-S populations. (**E**) Quantitative expression of *MpPLs* by qRT-PCR in the adult females of the THG-R and FFJ-S populations. The expression levels were normalized to β-actin genes. The significant differences are marked by asterisks: * at the 0.05 level; ** at the 0.01 level; *** at the 0.001 level.

In an analysis of the gene conservation regions in the aphid genome, a total of 12 phospholipase (PL) genes were identified. A phylogenetic tree analysis revealed that these 12 *MpPL* genes belonged to five distinct categories (Figure 8A). The *PLCB2* category contained the highest number of genes, with four genes, while *PLCB1*, *PLCD*, *PLCA2*, and *PLCABHD* each contained two genes (Figure 8A). The smallest number of motifs in these 12 *MpPL* genes was found to be 15 (Figure 8B). An analysis of the gene domains showed that the 12 genes collectively possessed 12 domains, namely, “Phospholip_B”, “Phospholip_B superfamily”, “PFU”, “PUL”, “WD40”, “Phospholipase_B_like”, “DDHD”, “WWE”, “Abhydrolase superfamily”, “Patatin_and_cPLA2 superfamily”, “ANKYR”, and “YheT” (Figure 8A). Ten of the genes contained only one domain.

The transcriptomic data of *M. persicae* revealed the transcriptional expression profiles of 12 *MpPL* genes, indicating that *MpPLBr*, *MpPLB1a*, *MpPLA2p*, *MpPLA2*, *MpPLDDH2*, *MpPLA2B*, and *MpPLABHD3b* exhibited higher expression levels, whereas *MpPLBr2* showed a lower expression level. The transcriptomic data also demonstrated that the expression levels of the *MpPLC* genes were lower in the THG-R population than in the FFJ-S population (Figure 8C). The quantitative fluorescence PCR results further confirmed that the expression levels of all 12 *MpPLC* genes were lower in the THG-R population than in the FFJ-S population. Specifically, in the second-instar nymphs of the THG-R population, the expression levels of *MpPLB1a*, *MpPLA2p*, *MpPLA2*, *MpPLDDH2*, *MpPLA2B*, *MpPLABHD3b*, *MpPLB2a*, *MpPLDDHD1*, and *MpPLB1b* were significantly lower than those in the FFJ-S population. Additionally, in the female adults of the THG-R population, the expression levels of *MpPLBr*, *MpPLDDH2*, *MpPLA2B*, *MpPLB2a*, *MpPLDDHD1*, *MpPLB1b*, *MpPLABHD3a*, and *MpPLB2b* were significantly lower than those in the FFJ-S population. The differences in the expressions of the other *MpPLC* genes were not significant (Figure 8D,E).

We also searched for the prostaglandin H synthase (cyclooxygenase) (*COX*) genes in the *M. persicae* genome and detected a total of two (LOC111037985 and LOC111037852). However, the transcriptomic data indicated that the two *COX* genes were expressed at very low levels in the adult aphids (FPKM < 0.1), and the only prostaglandin E synthase (PGES) gene (LOC111031794) was significantly downregulated in the THG-R population. We did not detect any prostaglandin D synthase genes and did not pursue further studies on them (Supplementary Table S2).

### 2.7. Induction of Expression of Significantly Overexpressed Genes in THG-R via Neonicotinoids

**Figure 9:**
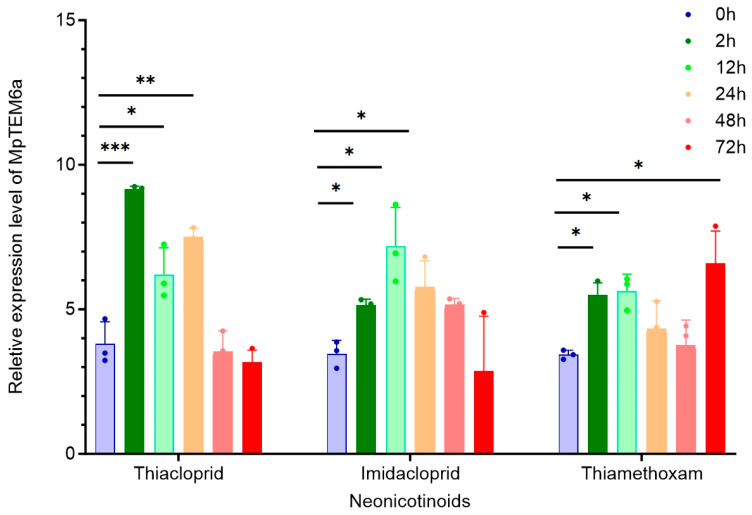
The mean expression level of selected *MpTEM6a* genes in the THG-R population at 0, 2 h, 12 h, 24 h, 48 h, and 72 h after the aphids were exposed to the recommended concentrations of thiacloprid (2270 mg L^−1^), imidacloprid (974 mg L^−1^), and thiamethoxam (36.5 mg L^−1^), compared to the FFJ-S population. The significant differences are marked by asterisks: * at the 0.05 level; ** at the 0.01 level; *** at the 0.001 level.

The above results indicate that *MpTEM6a* is significantly upregulated in the THG-G population. We also evaluated the induction of the expression of this gene after adult females were exposed to three neonicotinoids—thiacloprid, imidacloprid, and thiamethoxam—at the LC_50_ doses (Figure 9). The results showed that thiacloprid, imidacloprid, and thiamethoxam all significantly induced the expression of *MpTEM6a* in the THG-R population. The expression of *MpTEM6a* significantly increased at 2, 12, and 24h after treatment with thiacloprid and imidacloprid and at 2, 12, and 72h after treatment with thiamethoxam.

### 2.8. The Effect of MpTHEM6 Gene Knockdown on the Sensitivity of THG-R to Neonicotinoids

**Figure 10:**
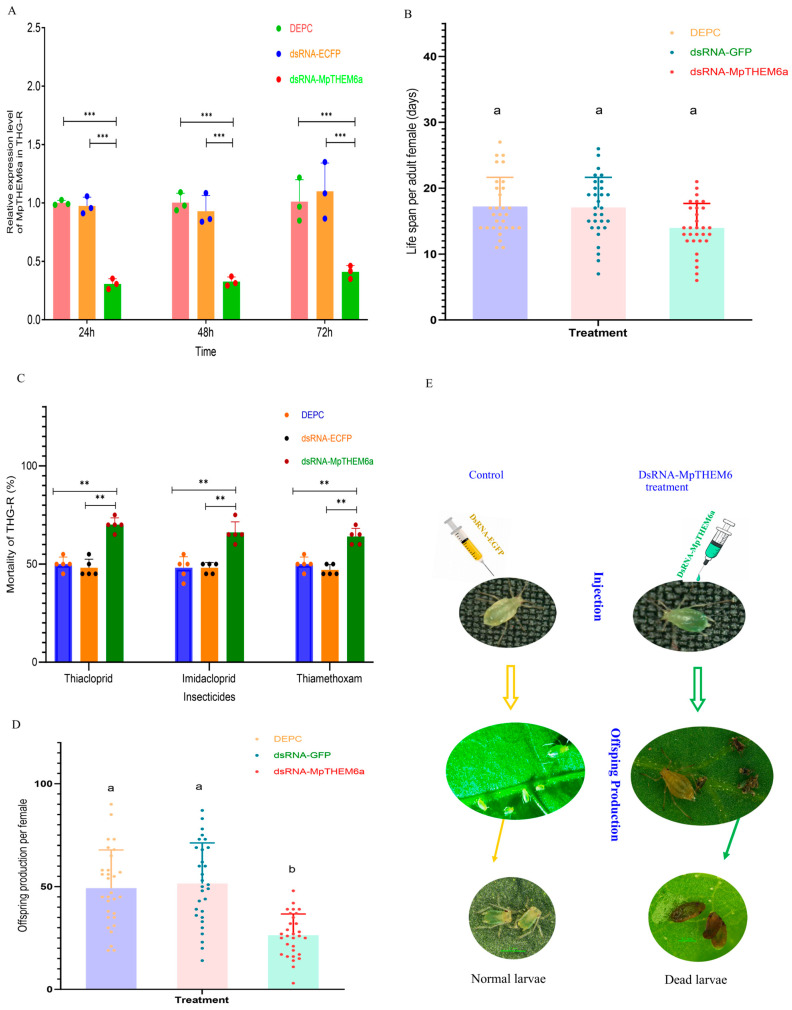
The effect of the knockdown of *MpTHEM6a* on the biological characteristics and the sensitivity of THG-R to neonicotinoids. (**A**) The relative expression levels of *MpTHEM6a*; (**B**) the effects of the knockdown of *MpTHEM6a* on adult female lifespan; (**C**) (%) mortality at 72 h of the THG-R population injected with dsRNA-MpTHEM6 after treatment with thiacloprid (2270 mg L^−1^), imidacloprid (974 mg L^−1^), and thiamethoxam (36.5 mg L^−1^); (**D**) the effects of the knockdown of MpTHEM6a on offspring production; (**E**) the effect of dsRNA-MpTHEM6 on offspring produced by the THG-R females. The significant differences are marked by asterisks: ** at the 0.01 level; *** at the 0.001 level. The bars with lowercase letters (a, b, c) are significantly different according to the one-way ANOVA, followed by Tukey’s multiple comparison test (*p* < 0.05).

To evaluate the functional roles of *MpTHEM6* in the resistance of THG-R to neonicotinoids, the MpTHEM6 gene was knocked down by RNA interference in this population, and the toxicity of thiacloprid, imidacloprid, and thiamethoxam was evaluated after RNAi. After fourth-instar THG-R larvae were injected with dsRNA-MpTHEM6, the transcript levels of MpTHEM6 at 24 h, 48 h, and 72 h significantly reduced by 0.31-, 0.33-, and 0.41-fold, respectively, compared with those of the control, which contained dsGFP (Figure 10A). Under the LC_50_ doses of thiacloprid, imidacloprid, and thiamethoxam, the mortality rates of the THG-R aphids injected with dsRNA-MpTHEM6 were 70%, 66%, and 64%, respectively, which were significantly higher than those injected with DEPC (50%, 48%, and 50%) and dsRNA-GFP (45%, 48%, and 47%) (Figure 10C).

### 2.9. Effect of MpTHEM6 Gene Knockdown on Adult Longevity and Offspring Production

The adult longevity of *M. persicae* and the fecundity per female at 21 °C were investigated after RNAi of *MpTHEM6*. Compared with FFJ-S, the apterous adult aphids of THG-R had a significantly reduced lifespan and a notable decrease in nymph production. The THG-R apterous adults treated with dsRNA-MpTHEM6 had a significantly shorter longevity than those treated with DEPC or dsRNA-GFP (Figure 10B). Additionally, the THG-R aphids treated with dsRNA-MpTHEM6 had the lowest fecundity (26.3 offspring), significantly lower compared to those treated with DEPC (49.3 offspring) or dsRNA-GFP (51.5 offspring) (Figure 10D). As shown in Figure 8E, the THG-R apterous adults injected with dsRNA-MpTHEM6 produced many nonviable nymphs, leading to a significant reduction in adult fecundity.

## 3. Discussion

The rapid development of metabolomics has laid the foundation for the rapid and accurate identification of insect metabolites. In this study, we utilized UHPLC-MS/MS non-targeted metabolomics techniques to identify lipid metabolites in adult female peach aphids. We identified a total of 148 lipid metabolites; this number is significantly higher than that found in adult fruit flies analyzed using LC-MS/MS (78 metabolites) [19] and in the fireflies *Aquatica leii* and *Lychnuris praetexta* analyzed using UHPLC-MS/MS (53 metabolites) [20]. However, it is lower than that found using high-resolution shotgun mass spectrometry in fruit flies across 27 developmental stages and raised on four different diets (250 metabolites). This discrepancy may be due to our analysis focusing solely on one insect stage, the adult stage of female aphids. The lipids that we identified belonged to 6 major classes and 15 subclasses, which is more than the 5 major classes (prenol lipids, steroids and steroid derivatives, fatty acyls, sphingolipids, and glycerophospholipids) found in fireflies [20].

The primary role of glycerophospholipids is to constitute the cellular membranes in all organisms and subcellular organelles, including PC, LPC, PE, LPE, PS, and PI. These cell membranes are composed of phospholipid bilayers, where the hydrophobic fatty acid chains face inward toward each other, while the hydrophilic polar head groups are positioned outward, interacting with the aqueous environment. The lipid composition of the honeybee brain is predominantly composed of glycerophospholipids, which make up approximately 88.89% of the total lipids [21]. Additionally, we identified a total of 84 glycerophospholipid species in *M. persicae*, including 28 PC species and 21 PE species, indicating a greater abundance of PC in the aphid’s body. Gao et al. [22] identified a total of 248 glycerophospholipid metabolites in *Aphis gossypii* parasitized by *Lysiphlebia japonica*. However, in insect neural cells, such as in the fruit fly’s membrane, PE is more prevalent than PC [23], and the PE content is greater than the PC content; for example, in the brain of bees, the PE content is 38.44%, while the PC content is 19.03% [21]. Insects differ from mammals in that this ratio is reversed in mammalian cells [24].

In the THG-R population, the majority of PE and LPE metabolites, as well as PC and LPC metabolites, exhibited significantly elevated levels compared with the FFJ-S population. As the current study did not examine alterations in the metabolite profiles of the peach aphid following treatment with neonicotinoid insecticides on the THG-R strain, it is impractical to ascertain whether this discrepancy is correlated with the enhanced resilience of *M. persicae* to high concentrations of neonicotinoids. Nevertheless, when *A. mellifera* are exposed to sublethal doses of neonicotinoid insecticides [21], there is a notable increase in the concentrations of various PE and PC metabolites in their brains, such as LPE 18:1, PC (18:1/18:1), and LPE 18:0. Furthermore, the rise in brain LPE 18:0e levels after exposure to neonicotinoid treatment in bees has been implicated in the induction of intense self-grooming behaviors.

In insects, the polyunsaturated fatty acid arachidonic acid (AA) is used to synthesize eicosanoids, which play several key roles in insect physiology and immunology, and its metabolic pathway is called the AA pathway. We found a significant difference between the THG-R and FFJ-S populations in terms of AA and its metabolites, such as PGs, EET, and HETE. Prostaglandins (PGs) are essential in modulating various facets of insect reproduction, encompassing oocyte development and oviposition-related behaviors, and several prostaglandins (PGs) in the THG-R population were found to be much higher than in the FFJ-S population. Insect tissues can produce a broad variety of PGs. Destephano et al. [25] confirmed that PGE2 production occurs in the male reproductive tract of *Acheta domesticus*. Using radioimmunoassays, Murtaugh and Denlinger [26] measured the relative amounts of PGE2 and PGFα (PGFα) in six distinct insect species. It was discovered that the hemocytes and fat bodies of *Manduca sexta* larvae can biosynthesize several PGs, such as PGA2, PGE2, PGD2, and PGF2α [27,28]. A total of nine prostaglandin compounds were discovered in *M. persicae*, namely, 16,16-Dimethyl PGA1, 8-iso PGA2, 15-Deoxy-Δ12,14-PGA1, PGB1, PGE2, PGE1, 6-keto-prostaglandin f1alpha, PGG2, and PGF3α, and they are involved in the body’s natural defense mechanisms. Nonetheless, PGB1 and PGG2, which have not been documented in other insects, were found to be elevated in the THG-R population. The literature indicates that PGB1 is a metabolite of PGA1 and an inhibitor of PLA2 activity [29,30]. Recently, it has been found that PGB1 remarkably increases in response to abiotic stress in some organisms. A metabolomic analysis revealed that the notable elevation in the differential metabolic markers PGB1 and AA facilitates marine shellfish larvae in acclimating to various artificial light at night (ALAN) conditions [31]. PGB1 was also found to be highly expressed in the urine of rats treated with high iA (100 mg/L NaAsO_2_) [32]. PGD2 and PGH2 were not detected in the adults of *M. persicae*. Tetranor-PGDM is a metabolite of PGD2, and due to its relatively stable chemical properties, it has been widely used as a biomarker for human disease diagnosis [33]. In this study, the level of tetranor-PGDM in the THG-R population was significantly lower than that in the FFJ-S population. However, the relationship between this low level and the aphids’ resistance to neonicotinoid insecticides requires further investigation.

Moreover, PGs can influence gene expression. Stanley and colleagues [34,35] found that PGA1, PGA2, and PGE1 can modulate gene expression in *Helicoverpa zea* cells, with 15 mM of PGA1 and PGE1 considerably enhancing the expression of HSP genes (heat shock proteins). In our recent analysis comparing transcriptomes, it was found that 28 out of 29 HSP genes in THG-R had reduced expression relative to those in FFJ-S (unpublished); this is potentially linked to the significant downregulated levels of PGA1 and PGE1 in THG-R compared with in FFJ-S. PGE2, among several PGs, has been extensively researched in insects and is crucial for numerous physiological activities, including reproduction, fluid secretion, aging, and immunological responses [36,37]. It has been determined to govern oviposition in *S. exigua* [38]. PGE2 plays a role in various aspects of ovarian development in female insects. The *mPGES2* gene in *D. melanogaster* has been shown to affect male fertility [39]. PGE2 is also involved in egg formation in specific species, including *Rhodnius prolixus* [40]. In this investigation, the PGE2 levels in THG-R were considerably lower than in FFJ-S. This study found that the egg-laying capacity of THG-R female adults was significantly inferior to that of FFJ-S female adults, potentially due to the reduced levels of PGE2.

Figure 1 shows the main enzymes involved in lipid metabolism. We identified their genes in the aphid genome based on their structural characteristics and found 11 *MpFAS* genes, 16 *MpELO* genes, 12 *MpFAD* genes, 12 *MpTE* genes, 22 *MpFAR* genes, and 12 MpPL genes. Due to space limitations, we did not construct a phylogenetic tree of these genes with related genes in other insects. The number of these genes in aphids differs significantly from that in other insects. From previous reports, it is known that the number of FAS genes in other insects, such as *Ae. aegypti* (five *FASs*), *D. melanogaster* (five FASs), *A. mellifera* (two *FASs*), and *Locusta migratoria* (two *FASs*), is lower than that in *M. persicae* [41,42]. The number of ELO genes in *M. persicae* is lower than that in *D. melanogaster* (20 *ELOs*) and *Tribolium castaneum* (18 *ELOs*) but higher than that in Tenebrio molitor (2 ELOs) [43]. The number of FAD genes in *M. persicae* is not significantly different from that in other insects, such as *Acromyrmex echinatior* (15 *FADs*), *Acyrthisiphon pisum* (13 *FADs*), and *D. melanogaster* (10 *FADs*) [44].

The expression levels of all *MpFASs*, *MpELOs*, *MpFADs*, and *MpPLs* in the second-instar nymphs and adults of THG-R were not significantly higher than those of the related genes in FFJ-S, which suggests that these genes may not directly participate in the resistance of *M. persicae* to neonicotinoids. They may be indispensable for insect development; in insects, gene expression leading to lipid accumulation can affect growth and development [45]. A previous study found that compared with a dsGFP injection group, the survival rate of *S. Litura* larvae decreased sharply after RNAi of the SlFAS1 gene [46]. Yang et al. [42] reported that knocking down the FAR genes LmFAS1 and LmFAS3 led to approximately 80% mortality in migratory locusts. In *Tenebrio molitor*, the RNAi silencing of TmELO1 led to an increase in mortality [43], and in D. melanogaster, RNAi of the ELO gene CG6660 also resulted in a similar lethal phenotype [47], indicating that ELO is indispensable for insect survival. These genes are vital for maintaining the normal growth and development of insects; knocking down several FADs in *N. Lugens* nymphs significantly increased nymph mortality [44]. Compared with FFJ-S, the lifespan and fertility of THG-R significantly decreased; this may be the “fitness cost”, and it may be caused by the significantly reduced expression of these genes in THG-R. However, further experiments are needed to verify the specific effects on the biology of neonicotinoid-resistant *M. persicae* in terms of certain lipid-related genes.

We found that the AA content in the THG-R population was much higher than in the FFJ-S population, indicating that the AA metabolic pathway in THG-R can help us determine the reasons for this metabolic difference (Figure 1). Studies on the AA pathway in mammals have shown that there are three pathways for AA synthesis: one is the hydrolysis of esterified AA on the inner surface of the cell membrane by phospholipase A2 (*PLA2*) into a free form. Additionally, PLA2 involvement in AA synthesis has been widely reported. We identified 12 PL genes in *M. persicae*, comprising 5 MpPLA2 genes, 5 *MpPLB* genes, and 2 *MpPLD* genes. It has been reported that in bacteria, *PLB* genes have the function of PLA2 genes [48]. However, compared with the FFJ-R population, the expression of these PL genes in the THG-R population was significantly downregulated or not significantly different, indicating that the rise in AA levels in the THG-R population is not caused by the overexpression of *PLA2*. In addition, AA can also be generated through the hydrolysis of the arachidonyl-CoA (AA-CoA) pathway through thioesterases, and acyl-CoA thioesterase 7 (*ACOT7*) is a key enzyme in humans for the hydrolysis of arachidonyl-CoA (AA-CoA) to generate AA [49,50,51]. However, this pathway has not been reported in insects. We identified 13 thioesterases in the peach aphid, of which 5 similar to ACOT7 displayed acyl-CoA thioesterase activity. However, none of these thioesterases were significantly overexpressed in THG-R. We detected the overexpression of a thioesterase gene, MpTHEM6a, in THG-R. Although lacking a defined biological role, THEM6 has recently been categorized in the thioesterase superfamily because of the presence of a “HotDog” domain, an evolutionarily conserved region anticipated to exhibit thioesterase activity [52].

The *THEM6* gene has not been previously reported in insects, and its association with insecticide resistance in pests has also not been previously reported. In this study, in addition to being expressed at higher levels in the THG-G population than in the FFJ-S population, it was also found that the overexpression of the *THEM6* gene in peach aphids could be induced by thiamethoxam, imidacloprid, and clothianidin. Recently, the human *THEM6* gene has become a research hotspot, as its expression is higher in colorectal, gastric, and breast cancer tissues than in normal tissues, and it has been considered a potential biomarker for these cancers [53,54,55]. The overexpression of *THEM6* has also been shown to promote the growth and migration of prostate cancer [56]. Functional studies found that knocking out *THEM6* inhibits tumor growth [57] and that high levels of THEM6 are associated with poor clinical outcomes and elevated UPR activation levels. These studies also demonstrated a significant association between high *THEM6* levels and high Ki67 expression in two groups of prostate cancer patients, indicating that *THEM6* is highly expressed in highly proliferative tumors [56,57]. *THEM6* is capable of regulating lipid metabolism, and knocking out the *THEM6* gene in 22rv1 cells leads to profound remodeling of the cellular lipidome. THEM6 depletion is associated with a significant reduction in intracellular levels of various triglycerides (TGs) and ether lipid species, including ether TGs, ether PCs, and ether PEs. In contrast, the number of ceramides in *THEM6* knockout cells increases, and, in addition to causing specific lipid changes, knocking out *THEM6* also significantly affects the total amounts of TGs, ether TGs, and ceramides in 22rv1 cells [57]. In this study, when we knocked down *MpTHEM6a*, the toxicity of the neonicotinoid insecticides significantly increased, but this did not affect the lifespan of the adults. Further research is needed to understand how *MpTHEM6a* increases aphid resistance to neonicotinoid insecticides by regulating lipid synthesis.

## 4. Materials and Methods

### 4.1. Insects

Two *M. persicae* populations (FFJ-S and THG-R) were used in this study. The FFJ-S population was susceptible to neonicotinoids, and the LC_50_ values of this population for thiacloprid (97.5% purity; Bayer AG, Leverkusen, Germany), imidacloprid (97% purity; Bayer AG, Germany), and thiamethoxam (98% purity; Syngenta Group, Dielsdorf, Switzerland) were 1.89, 1.09, and 2.57 mg L^−1^, respectively [5]. The THG-R strain was established from the FFJ-S strain via successive screening with thiacloprid for more than 50 generations in the laboratory, and the LC50 values of this population for thiacloprid, imidacloprid, and thiamethoxam were 2270, 974, and 36.5 mg L^−1^, with a 1200-, 890-, and 11-fold resistance when compared with FFJ-S. Both GPA strains were reared on pepper seedlings, *Capsicum annuum* L., under controlled conditions of 19–22 °C, a 60% relative humidity, and a photoperiod of 16:8 h (light–dark).

### 4.2. Metabolite Extraction and Analysis

Approximately 1200 apterous female adults were collected from the FFJ-S and THG-R strains. Each sample consisted of 200 aphids (six replicates), which were individually ground with liquid nitrogen, and the resulting homogenate was resuspended in pre-chilled 80% methanol using a vortex mixer. The samples were then incubated on ice for 5 min and centrifuged at 15,000× *g* and 4 °C for 20 min. A portion of the supernatant was diluted to a final concentration with 53% methanol using LC-MS-grade water. The diluted samples were transferred to new Eppendorf tubes and centrifuged again at 15,000× *g* and 4 °C for 20 min. The final supernatant was then analyzed using the LC-MS/MS system, as described by Want et al. [58].

UHPLC-MS/MS analyses were conducted using a Vanquish UHPLC system (Thermo Fisher, Lenexa, KS, USA) coupled with an Orbitrap Q Exactive^TM^ HF mass spectrometer (Thermo Fisher, Lenexa, KS, USA) at Novogene Co., Ltd. in Beijing, China. The samples were injected into a Hypesil Gold column (100 × 2.1 mm, 1.9 μm) and separated using a 12 min linear gradient at a flow rate of 0.2 mL/min. For the positive polarity mode, the mobile phase consisted of eluent A (0.1% formic acid in water) and eluent B (methanol). In the negative polarity mode, eluent A was 5 mM ammonium acetate at pH 9.0, and eluent B was methanol. The solvent gradient was programmed as follows: starting at 2% B for 1.5 min, ramping up to 85% B over 3 min, holding at 100% B for 10 min, returning to 2% B in 0.1 min, and finally maintaining at 2% B for 12 min. The Q Exactive^TM^ HF mass spectrometer was operated in both positive and negative polarity modes with a spray voltage of 3.5 kV, a capillary temperature of 320 °C, a sheath gas flow rate of 35 psi, an auxiliary gas flow rate of 10 L/min, an S-lens RF level of 60, and an auxiliary gas heater temperature of 350 °C [59,60].

The initial data files, produced by Ultra-High Performance Liquid Chromatography–Mass Spectrometry/Mass Spectrometry (UHPLC-MS/MS), underwent processing with Compound Discoverer 3.1 (CD3.1, Thermo Fisher) for the purpose of peak alignment, detection, and quantification of each metabolite. The key parameters were configured as follows: a retention time tolerance of 0.2 min; an actual mass tolerance of 5 ppm; a signal intensity tolerance of 30%; a signal-to-noise ratio of 3; and a minimum intensity threshold, among others. Subsequently, peak intensities were normalized relative to the overall spectral intensity. This normalized data were then utilized to predict the molecular formulae by analyzing adduct ions, molecular ion peaks, and fragment ions. Following this, the peaks were correlated with the mzCloud (https://www.mzcloud.org/, accessed on 11 May 2024), mzVault, and MassList databases to achieve precise qualitative and relative quantitative outcomes. Statistical evaluations were conducted using the statistical software R (version R3.4.3), Python (version 2.7.6), and CentOS (version 6.6). In instances where data did not exhibit a normal distribution, normalizations were attempted through the application of an area normalization method [61,62].

### 4.3. A Preliminary Search and Identification of Key Lipid Metabolic Enzymes in the M. persicae Genome

We used a keyword search, the Hidden Markov Model (HMM), and the Basic Local Alignment Search Tool (BLAST) to search for six enzymes related to lipid metabolism in peach aphids, namely, fatty acid synthase (FAS), the elongase of very-long-chain fatty acids (ELO), fatty acid desaturase (FAD), thioesterase (TE), and phospholipase (PL). The genes for these lipid metabolic enzymes in *M. persicae* are abbreviated as follows: “*MpFAS*” for fatty acid synthase, “*MpELO*” for the elongase of very-long-chain fatty acids, “*MpFAD*” for fatty acid desaturase, “*MpTE*” for thioesterase, and “*MpPL*” for phospholipase. For genes that already had assigned names in NCBI, we used those existing names. Preliminary data on these enzymes were collected using the following steps: (1) A keyword search was conducted for the above-mentioned enzymes in the *Myzus persicae* database (https://bipaa.genouest.org/sp/myzus_persicae_g006/, accessed on 8 April 2024). (2) The heme peroxidase protein-conserved domain model (*MpFAS*: PF14765; *MpELO*: PF01151; *MpFAD*: PF00487; *MpTE*: PF13279 and PF02338; *MpPL*: PF12796 and PF09070) was downloaded from the Pfam library (http://pfam.xfam.org/, accessed on 12 April 2024) using HMMER 3.4_Windows software (http://hmmer.org/, accessed on 12 April 2024) [63]. (3) The *M. persicae* database (https://bipaa.genouest.org/sp/myzus_persicae_g006, accessed on 13 April 2024) was accessed to retrieve the gene protein sequences, followed by the application of the BLASTP method and the elimination and combination of duplicated genes.

### 4.4. The Construction of a Phylogenetic Tree and a Protein Domain Analysis of the Lipid Metabolic Enzymes for M. persicae

The multiple-sequence alignments of the six enzyme proteins in *M. Persicae* were analyzed via MEGA 11.0 software using the Muscle algorithm. Then, the neighbor-joining (NJ) method was employed, along with 1000 bootstraps, to construct an evolutionary tree. The remaining parameters were set to their default values [64,65,66].

### 4.5. The Transcriptome Profiles of the THG-R and FFJ-S Populations

The total RNA from approximately 600 apterous adult aphids per treatment, with a cumulative total of 1800 aphids across three biological replicates, was isolated using the TRIzol^TM^ reagent (Invitrogen, Carlsbad, CA, USA) in accordance with the manufacturer’s specified protocol. The subsequent steps of RNA purification, cDNA synthesis via reverse transcription, library construction, and high-throughput sequencing were conducted at Shanghai Majorbio Bio-pharm Biotechnology Co., Ltd. (Shanghai, China), following the standardized procedures provided by the service provider. The sequencing library was prepared on the state-of-the-art NovaSeq X Plus platform (PE150) with the corresponding NovaSeq Reagent Kit. We subsequently performed a comparative analysis of the transcriptomic profiles of the selected genes across the distinct treatment conditions to elucidate differential gene expression patterns. To discern the differential expression of the six lipid metabolic enzyme genes between the THG-R and FFJ-S populations, we quantified the expression level of each transcript using the FPKM (fragments per kilobase of transcript per million fragments mapped) metric. RSEM (RNA-Seq by Expectation Maximization) was employed for the estimation of gene abundance. A differential expression analysis was conducted using either DESeq2 or DEGseq [67,68].

### 4.6. Quantitative Real-Time PCR Analysis of Different Enzyme Genes in Both THG-R and FFJ-S Populations and Expression Induction of Selected Upregulated Genes in THG-R Population via Nicotinoid Exposure

The expression levels of the six enzyme genes in the treated aphids were quantified using a reverse transcription quantitative polymerase chain reaction (RT-qPCR) with SYBR^®^ Green Supermix (Thermo Fisher, Waltham, MA, USA) on a qTOWER 2.2 real-time PCR system (Analytikjena, Jena, Germany). Total RNA extraction and quantification were performed as previously described, using a ScanDrop 100 spectrophotometer (Analytikjena, Jena, Germany) in accordance with the manufacturer’s instructions. RNA was diluted to a concentration of 0.8 μg/μL with diethyl pyrocarbonate (DEPC)-treated water, and 0.8 μg of RNA was reverse-transcribed in a 20 μL reaction volume using a TUREscript 1st Strand cDNA Synthesis Kit (Aidlab, Beijing, China), with the actin gene serving as an internal control (NCBI gene ID: 836110). Each RT-qPCR reaction consisted of a 20 μL mixture comprising 1 μL of sample cDNA, 1 μL of each primer at a concentration of 200 nM, 6 μL of DEPC-treated water, and 10 μL of 2 × SYBR^®^ Green Supermix. The qPCR cycling conditions were as follows: initial denaturation at 95 °C for 3 min, followed by 39 cycles of denaturation at 95 °C for 10 s, and annealing/extension at 58 °C for 30 s. A plate reader was employed for data analysis. A melting curve analysis was conducted from 60 °C to 95 °C. Primers for these genes were designed using Primer Express 3.0 software, based on the target gene sequences available in the NCBI database, and they are provided in Supplementary Table S1.

Meanwhile, the significantly upregulated genes in THG-R were selected for expression induction studies. THG-R apterous adults were transferred to pepper leaves treated with LC_50_ doses of thiacloprid (2270 mg L^−1^), imidacloprid (974 mg L^−1^), and thiamethoxam (36.5 mg L^−1^) for 2, 12, 24, 48, and 72 h to determine the effects of thiacloprid on the expression of significantly upregulated genes. The experiment included three replicates, and each replicate contained 30 adults. The insects were frozen in liquid nitrogen and stored at −80 °C. The relative gene expression was calculated automatically using qPCRsoft 3.2 software. The relative gene expression 2^−ΔΔCt^ method was used to calculate the relative fold gene expression of the samples [69].

### 4.7. RNA Interference

MpTHEM6a double-stranded RNAs (dsRNAs) were obtained by using a T7 high-yield transcription kit (Invitrogen, USA) in accordance with the manufacturer’s instructions. The primers utilized for dsRNA synthesis are listed in Supplementary Table S1. A total of 20 ng/μL of the dsRNA that targeted the desired gene was injected into the apterous adults with a Nanoject III^TM^ nanoliter injector (Drummond Scientific Company, Broomall, PA, USA). DEPC and dsRNA-EGFP were employed as controls [5]. After the injection, the aphids were transferred to pepper seedings. Three biological replicates were used in the experiment, and each included thirty aphids. RT-qPCR was used to assess the efficacy of the dsRNA in suppressing the expression of the MpTHEM6a genes after 72 h.

To assess the susceptibility of *M. persicae* to thiacloprid, imidacloprid, and thiamethoxam following RNA interference (RNAi) targeting of MpTHEM6a, we administered the recommended doses of these three neonicotinoids to the apterous adults 48 h post-treatment. Control groups consisted of adults treated with DEPC water and dsRNA-GFP. The mortality of *M. Persicae* was evaluated 48 h after neonicotinoid treatment. Furthermore, we monitored the longevity of the adults and the fertility of each female following the RNAi treatment. The injected adults were then placed on pepper seedlings to determine the duration of survival and offspring production until their deaths. Mortality assays were replicated five times, with ninety treated apterous adults used for the analysis of adult longevity and fecundity.

## 5. Conclusions

Through a comparative metabolomics analysis, we found significant differences between THG-R and FFJ-S in terms of lipid metabolites, mainly phospholipids and fatty acids. The metabolites related to AA metabolic pathways, such as AA and prostaglandin compounds, showed considerable differences. In THG-R, AA, (±)11-HETE, and prostaglandin B1 were significantly upregulated, while prostaglandin A1, tetranor-PGDM, 8,15-diHETE, and (±)11(12)-EET were significantly decreased, and these metabolites could all serve as biomarkers. To further clarify the causes of these differences, we selected several major key enzymes involved in the metabolic process of fatty acid synthesis and used transcriptomic methods to determine the differences in the expression of these enzymes between the THG-R and FFJ-S populations. The results showed that most of the metabolic enzymes selected in THG-R were not overexpressed. However, the MpTHEM6a gene was significantly upregulated in THG-R. The overexpression induced by neonicotinoid insecticides and the enhanced effects on neonicotinoid insecticides observed in the RNAi experiments both suggest that MpTHEM6a is associated with the resistance of peach aphids to neonicotinoid insecticides.

## Data Availability

All data are provided in the paper or in the Supplementary Materials.

## Supplementary Materials

The following supporting information can be downloaded at: https://www.mdpi.com/article/10.3390/ijms252212112/s1.
