# Supplementary figures and images for: The Altered Lipid Composition and Key Lipid Metabolic Enzymes in Thiacloprid-Resistant Myzus persicae, with Special Attention Paid to the Function of MpTHEM6a
Source: Int J Mol Sci. 2024 Nov 11;25(22):12112. doi: 10.3390/ijms252212112 (PMC11594901; doi:10.3390/ijms252212112)

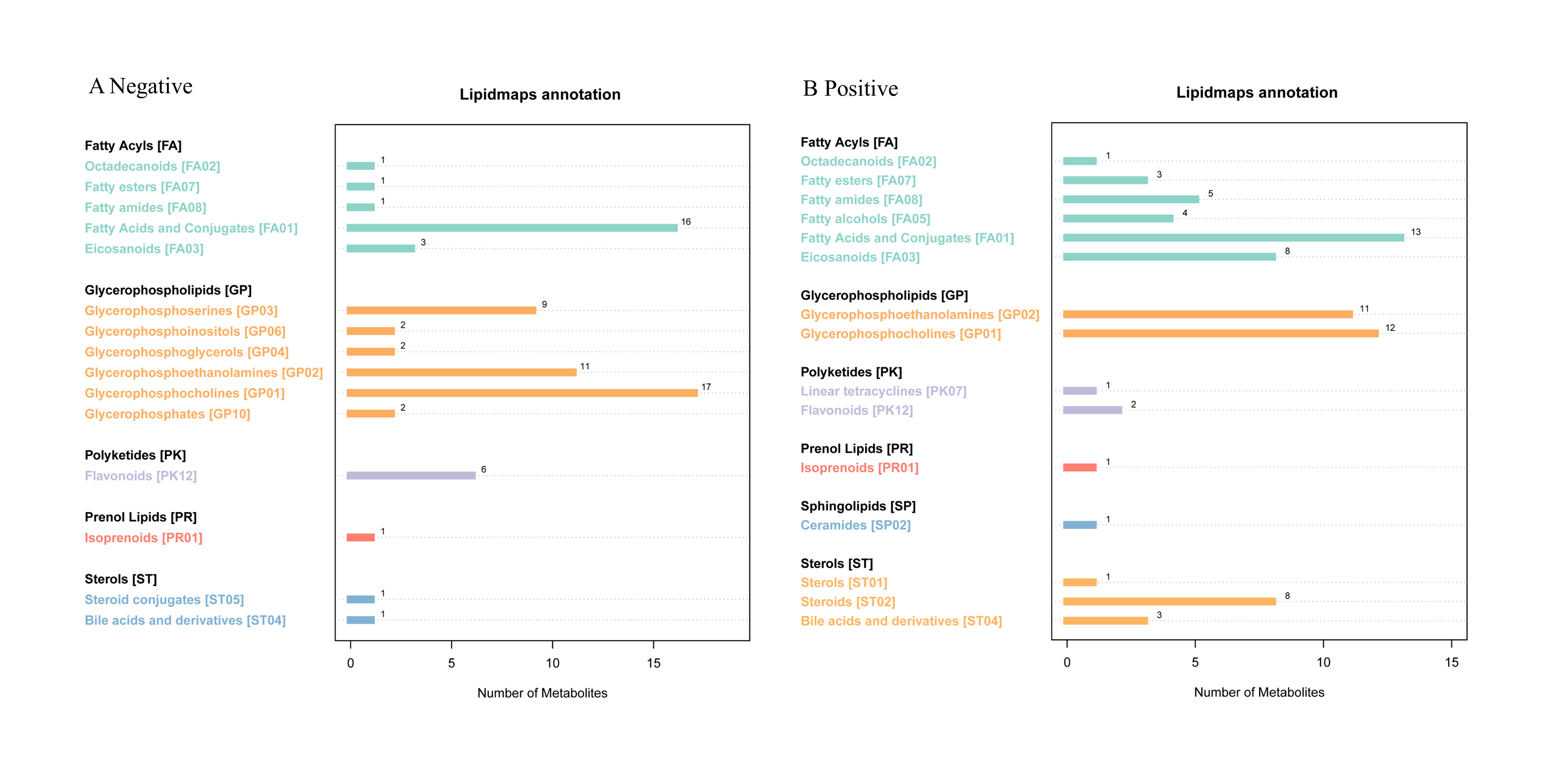

Supplement: Supplementary file 1 [file ijms-25-12112-s001.zip › Figure S1 Lipid_category.tif]
